# Reduced Representation Bisulfite Sequencing Determination of Distinctive DNA Hypermethylated Genes in the Progression to Colon Cancer in African Americans

**DOI:** 10.1155/2016/2102674

**Published:** 2016-09-01

**Authors:** Hassan Ashktorab, Afnan Shakoori, Shatha Zarnogi, Xueguang Sun, Sudhir Varma, Edward Lee, Babak Shokrani, Adeyinka O. Laiyemo, Kareem Washington, Hassan Brim

**Affiliations:** ^1^Department of Medicine and Cancer Center, Howard University, Washington, DC, USA; ^2^Department of Genetics, Howard University, Washington, DC, USA; ^3^Umm AL-Qura University, Makkah, Saudi Arabia; ^4^DNA Sequencing and Genotyping Core, Cincinnati, OH 45229, USA; ^5^Hithru Analytics, Laurel, MD, USA; ^6^Department of Pathology, Howard University, Washington, DC, USA

## Abstract

*Background and Aims*. Many studies have focused on the determination of methylated targets in colorectal cancer. However, few analyzed the progressive methylation in the sequence from normal to adenoma and ultimately to malignant tumors. This is of utmost importance especially in populations such as African Americans who generally display aggressive tumors at diagnosis and for whom markers of early neoplasia are needed. We aimed to determine methylated targets in the path to colon cancer in African American patients using Reduced Representation Bisulfite Sequencing (RRBS).* Methods*. Genomic DNA was isolated from fresh frozen tissues of patients with different colon lesions: normal, a tubular adenoma, a tubulovillous adenoma, and five cancers. RRBS was performed on these DNA samples to identify hypermethylation. Alignment, mapping, and confirmed CpG methylation analyses were performed. Preferential hypermethylated pathways were determined using Ingenuity Pathway Analysis (IPA).* Results*. We identified hypermethylated CpG sites in the following genes:* L3MBTL1, NKX6-2, PREX1, TRAF7, PRDM14*, and* NEFM* with the number of CpG sites being 14, 17, 10, 16, 6, and 6, respectively, after pairwise analysis of normal versus adenoma, adenoma versus cancer, and normal versus cancer. IPA mapped the above-mentioned hypermethylated genes to the Wnt/*β*-catenin, PI3k/AKT, VEGF, and JAK/STAT3 signaling pathways.* Conclusion*. This work provides insight into novel differential CpGs hypermethylation sites in colorectal carcinogenesis. Functional analysis of the novel gene targets is needed to confirm their roles in their associated carcinogenic pathways.

## 1. Background

Colorectal cancer (CRC) is the most common gastrointestinal cancer in the United States [1]. It is a major cause of cancer-related mortality [2]. CRC occurs through different molecular mechanisms and pathways, leading to different clinical and pathological outcomes. CRC occurs more frequently in African Americans (AAs) compared to any other racial group in the United States [1, 3, 4]. The reasons for this disparity include environmental factors as well as genetic specific predispositions [1]. It is now widely recognized that, in addition to genetic mutations, epigenetic mechanisms especially aberrant DNA methylation are involved in virtually every step of CRC development and progression.

DNA methylation is one of the most important epigenetic events that is thought to occur during the early stages of such oncogenic transformation [5]. CpG islands, the target for methylation, consist of short stretches of CpG rich regions that are often associated with promoter regions of genes [6]. In neoplastic cells, there is a modest global depletion of cytosine methylation but considerable acquisition of aberrant methylation within certain promoters' associated CpG islands [7]. This aberrant promoter methylation generally leads to epigenetic silencing of gene expression [7].

The aberrant methylation of CpG islands within gene promoters and/or first exonic/intronic regions is a recognized epigenetic event that leads to transcriptional silencing of corresponding tumor suppressor genes in CRC and other cancers. Regardless of the biological consequences of methylation-induced silencing of tumor suppressor genes, this epigenetic alteration constitutes a molecular signature that can serve as promising biomarkers for early detection [3, 8]. Many genes are silenced by aberrant methylation in CRC* (APC, p16INK4a*, and* TIMP3)*; such genes have the potential to become useful biomarkers for early detection of colorectal neoplasia [3, 8–10]. Early detection of colonic lesions is the most effective approach to reduce CRC incidence and mortality [11]. Therefore, molecular studies aimed at identifying CRC-specific methylation markers may provide useful insight for a better understanding of CRC progression [12, 13].

Most colorectal cancers arise from adenomatous polyps [14]. The progression to colorectal cancer is marked by specific ordered events that drive the “Adenoma-Carcinoma Sequence.” Methylation in an average of 14 genes is required for the formation of malignant tumors and fewer changes only suffice for benign tumorigenesis [15]. Even though genetic alterations occur according to a preferred sequence, the total accumulation of changes rather than their order is responsible for the tumors progression and behavior.

DNA methylation can target genes involved at different stages and in different cellular pathways. Promoter methylation of CHFR (checkpoint with fork-head and ring-finger domains) was found to be associated with survival and was considered to be an independent predictor for tumor recurrence. IGFBP3 (insulin-like growth factor binding protein 3) and CD109 DNA methylation were associated with worse survival for stage II CRC. Tumor suppressor genes (TSG) including* SOCS3, TFAP2E*, and* CDH4* have also been reported as DNA hypermethylation targets of which the gene expression was silenced in CRC tumors [16–19].

In this study, we investigated distinctive DNA methylation targets in normal, adenoma, and cancer specimens from African Americans using Reduced Representation Bisulfite Sequencing to detect novel DNA methylation targets in the path to cancer.

## 2. Materials and Methods

### 2.1. Patients and Tissue Samples

The samples were obtained from African American (AA) patients at Howard University Hospital (HUH) who underwent colonoscopy and/or surgery for the removal of colonic lesions (Table 1). All samples were analyzed by an expert gastrointestinal pathologist of the HUH Department of Pathology and kept frozen until used for DNA extraction. The samples consisted of a blood sample, a normal colon tissue, a tubular adenoma, a tubulovillous adenoma, and 5 cancers. These cancers were included to select genes that are consistently methylated at the cancer stage. Clinical, demographic, and pathological data were collected for all specimens (Table 1). Informed consent was obtained from all participants. This research was approved by Howard University Institutional Review Board (IRB 06-MED-39).

### 2.2. DNA Extraction

Genomic DNA was extracted from fresh frozen tissues. The samples were homogenized in lysis buffer consisting of 100 mM Tris-HCl (pH 8.5), 5 mM EDTA, 0.2% SDS, and 200 mM NaCl. Proteinase K was freshly added at a final concentration of 300 *μ*g/mL. Samples were incubated overnight at 55°C to ensure that genomic DNA is completely dissociated from any DNA binding proteins. After digestion, genomic DNA was extracted using a QIAGEN's genomic DNA extraction kit (Germantown, CA), according to the manufacturer's instructions (AllPrep QIAGEN kit). DNA, quality, and quantity were assessed using a NanoDrop spectrophotometer and 0.8% agarose gel electrophoresis.

### 2.3. EpiQuest Library Construction

EpiQuest libraries were prepared according to Zymo Research Protocol (Irvin, CA). 200–500 ng of genomic DNA were digested with NEB 60 units of* Taq*I and 30 units of* Msp*I (Ipswich, MA, USA) sequentially. Size-selected* Taq*I*-Msp*I fragments (40–120 bp and 120–350 bp) were filled-in and 3′-terminal-A extended, extracted with Zymo Research (ZR) DNA Clean and Concentrator*™*-5 kit (Irvin, CA). Ligation to preannealed adapters containing 5′-methyl-cytosine was performed using Illumina's DNA preparation kit and protocol (San Diego, CA). Purified adaptor ligated fragments were bisulfite-treated using the EZDNA Methylation-Direct*™* Kit (Irvin, CA). Preparative-scale PCR was performed. DNA Clean and Concentrator-purified PCR products were subjected to a final size selection on a 4% NuSieve 3 : 1 agarose gel. SYBR-green-stained gel slices containing adaptor-ligated fragments of 130–210 bp or 210–460 bp in size were excised. Library material was recovered from the gel (Zymoclean*™* Gel DNA Recovery Kit, Irvin, CA, USA) and sequenced on an Illumina HiSeq Genome Analyzer (San Diego, CA).

### 2.4. EpiQuest Sequence Alignments and Data Analysis

Sequence reads from bisulfite-treated EpiQuest libraries were identified using standard Illumina base-calling software and then were analyzed using a Zymo Research proprietary analysis pipeline according to the manufacturer's recommendations (Zymo Research, CA, USA). Residual cytosines (Cs) in each read were first converted to thymines (Ts), with each such conversion noted for subsequent analysis. A reference sequence database was constructed from the 50 bp ends of each computationally predicted* Msp*I*-Taq*I fragment in the 40–350 bp size range. All Cs in each fragment were then converted to Ts; the converted reads were aligned to the converted reference. The number of mismatches in the induced alignment were counted between the unconverted read and reference, ignoring cases in which a T in the unconverted read matched to a C in the unconverted reference. For a given read, the best alignment was kept if the second best alignment had 2 more mismatches; otherwise the read was discarded as nonunique. The methylation level of each sampled cytosine was estimated as the number of reads reporting a C divided by the total number of reads reporting a C or T. Fisher's exact test or *t*-test was used for each CpG site that has at least 5 reads covered. Also, promoter, gene body, and CpG island annotations were added for each CpG. The software pipeline is implemented in Python.

### 2.5. Bisulfite Conversion and Multiplex Amplification

Samples were subjected to sodium bisulfite treatment using the EZ DNA Methylation-Direct*™* Kit (Irvin, CA). Targeted amplification was done via Fluidigm 48, the 48 Access Array using Zymo Research's targeted sequencing service protocol. Sample loading, harvesting, and pooling were performed according to the manufacturer's protocol. Methylation profiling data from AA patients were compared with a normal candidate peripheral blood specimen as well as a normal colorectal tissue. A pairwise DNA methylation analysis was performed between colorectal neoplasia samples such as tubular adenoma, tubulovillous adenoma, and tumors versus normal tissue samples (blood and normal colon).

### 2.6. Barcoding/Adapterization PCR

Amplicon pools for each sample were diluted 1 : 100 and then amplified using barcoded adaptor-linkers received from Fluidigm according to the manufacturer's protocols (Fluidigm, San Francisco, CA). Reactions were cleaned up using the DNA Clean and Concentrator-5 and the products were normalized by concentration and pooled. Sequencing, alignment, and data analysis libraries were denatured, diluted, and sequenced on the Illumina MiSeq according to the manufacturer's protocols (San Diego, CA). The sequencing run was a 150-base paired-end run. Sequence reads were aligned and analyzed as described above.

### 2.7. Pathway Analysis

Further analysis was performed using the Ingenuity Pathway Analysis (IPA) software. The IPA “Upstream Regulator Analysis” predicts upstream regulators by combining the directional methylation changes from our RRBS targeted methylation-sequencing and knowledge from prior experimental reports on causal effects between molecules such as TSG and oncogenes, compiled in the IPA Knowledge Base. Upstream Regulator Analysis calculates a *Z*-score based on the edge of dysregulation of all the downstream molecules and the uniformity of the existing evidence about the upstream-downstream relation, for every upstream regulator known to have a causal effect on at least 4 (activating and inhibiting Up/Down) dysregulated genes/transcripts. *Z*-scores of <0 and >0, respectively, indicate a significant inhibition and activation state of the upstream regulator, regardless of the actual expression level of these molecules. See (http://pages.ingenuity.com/rs/ingenuity/images/0812%20upstream_regulator_analysis_whitepaper.pdf). The Network Generation Algorithm links molecules based on experimentally observed interactions and based on their interconnectedness. In general, the more interactions with other network members, the more central a molecule will be in a network.

## 3. Results

### 3.1. RRBS Global Methylation Analysis Revealed Candidate Genes

RRBS sequencing data from the 9 AA samples was obtained as described above and analyzed in pairwise fashion to determine differentially methylated CpG sites with relevance to colorectal neoplasia. This analysis showed increasing DNA methylation from normal to cancer with an average rate range from 0.09 (*L3MBTL1*) to 0.27 (*PRDM14*) (Table 2).

The RRBS analysis led to a list of genes that were differentially methylated in normal, tubular adenoma, tubulovillous adenoma, and colorectal neoplasia samples (Table 2). These genes (*L3MBTL1, NKX6-2, PREX1, TRAF7, PRDM14*, and* NEFM*) were methylated significantly in tumors when compared to normal tissue (*P* < 0.05).

The RRBS results for* L3MBTL1* (NM_032107) showed 22 methylated CpG sites within the promoter region in tumor samples. None of the CpG sites were methylated in normal, tubular adenoma, and tubulovillous adenoma samples (Tables 2 and 3).

As for* NKX6-2* (NM_177400), we identified 37 methylated CpG sites in promoter region in tumor samples, while none of these CpG sites were found to be methylated in normal, tubular adenoma, and tubulovillous adenoma samples (Tables 2 and 3).

For the* PREX1 *gene (NM_020820), we identified 27 methylated CpG sites in the promoter region in tumor samples and 6 methylated CpG sites in tubulovillous adenoma samples. None of these CpG sites were methylated in normal and tubular adenoma samples (Tables 2 and 3).

*TRAF7* (NM_032271) displayed differential methylation for 17 CpG sites in tumor samples, while none of these were methylated in normal, tubular adenoma, and tubulovillous adenoma samples (Tables 2 and 3).

The* PRDM14* (NM_024504) gene displayed methylation in 28 CpG sites in this gene's promoter region within tumor samples, while none of them was found to be methylated in normal, tubular adenoma, and tubulovillous adenoma samples (Tables 2 and 3).

**Figure 1:**
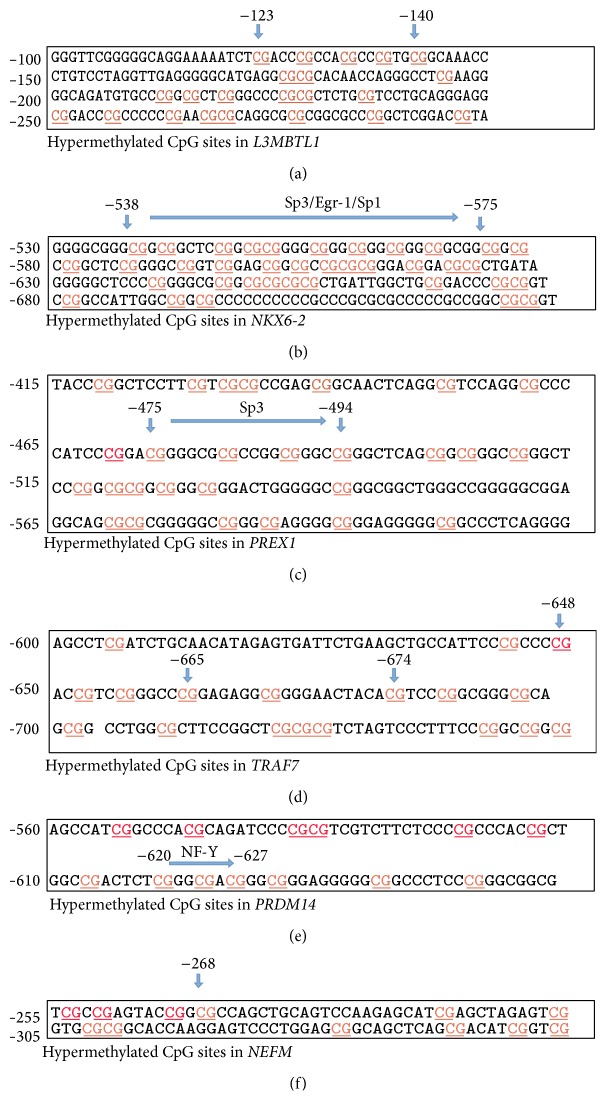
DNA methylation profile of RRBS analyzed hypermethylated genes in the tumor samples of African American patients:* L3MBTL1* (a),* NKX6-2* (b),* PREX1* (c),* TRAF7* (d),* PRDM14* (e), and* NEFM* (f).

The last identified marker in this study is* NEFM* (NM_005382) where 12 methylated CpG sites were identified within the gene's promotor region in tumor samples, while no CpG sites were found to be methylated in normal, tubular adenoma, and tubulovillous adenoma samples (Tables 2 and 3). Methylated CpG sites and their exact positions for the above genes are depicted in Figure 1.

### 3.2. Ingenuity Pathway Analysis

**Figure 2:**
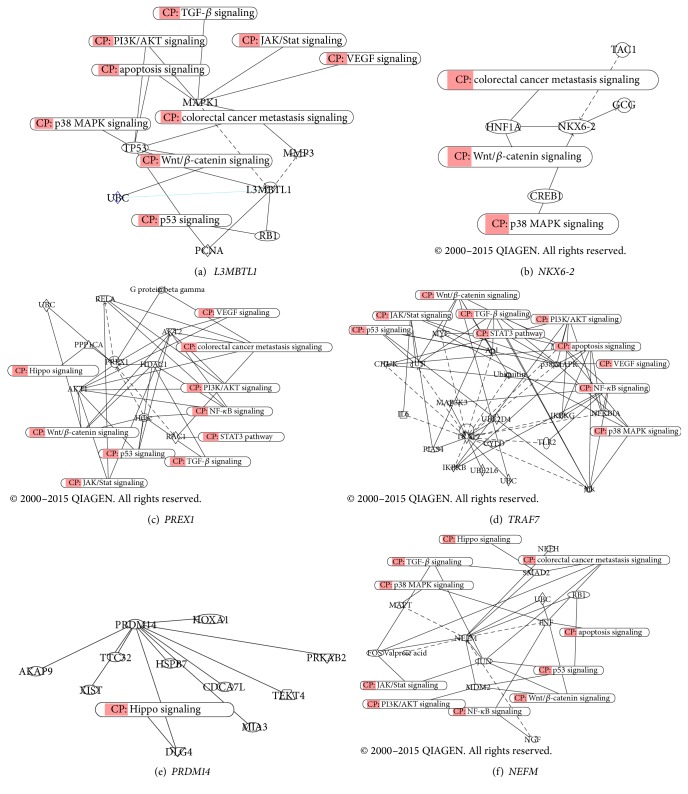
Ingenuity Pathways Analysis of the identified methylated targets.* L3MBTL1* (a),* NKX6-2* (b),* PREX1* (c),* TRAF7* (d),* PRDM14* (e), and* NEFM* (f). CP: Conical Pathway.

The hypermethylated markers were analyzed for their potential biological function(s) using the IPA software.* L3MBTL1, NKX6-2, PREX1*,* TRAF7, PRDM14*, and* NEFM* were mapped to Wnt/*β*-catenin, P53, TGF-*β*, PI3k/AKT, VEGF, NF-k*β*, Hippo, P38 MAPK, and JAK/STAT3 signaling pathways (Table 4) (Figure 2).

### 3.3. Pathway Analysis of Differentially Methylated Genes

Running the upstream and downstream regulator analysis led to the identification of 6 crosstalk regulators (Table 4), namely,* MMP3*,* GCG*,* HDAC1* and* AKT1*,* DKG4*, and S*MAD2* that are affected by* L3MBTL1*,* NKX6-2*,* PREX1*,* PRDM14*, and* NEFM*, respectively. Both endogenous and exogenous molecules are included in this analysis. All significant regulators which are classified as transcription factors are displayed in Table 4. All other regulator types can be found in Figure 2 as well. The significantly dysregulated molecules were sorted by their function or involvement in pathophysiological processes.

### 3.4. Network of Dysregulated Molecules Involved in IPA-Function

The following cellular functions have been potentially affected through the methylation of the identified 6 methylation targets (Figure 2).

*Apoptosis Response*.* L3MBTL1, PREX1, TRAF7, PRDM14*, and* NEFM* are part of apoptosis response since they interact with P53, Hippo, and P38 MAPK pathways. 

*Proliferation Response*.* L3MBTL1, PREX1, TRAF7*, and* NEFM* are part of proliferation response as they interact with TGF-*β* and Hippo pathways. 

*Inflammatory Response*.* PREX1, TRAF7*, and* NEFM *are part of inflammatory response because they interact with NF-k*β* pathway.

## 4. Discussion

Many approaches have been used to detect the status of DNA methylation in human tissues. Most of these methods relied on the preknowledge of the analyzed methylation targets such as* MLH1*,* p16*, and other known genes in colon neoplastic transformation. Herein, we used Reduced Representation Bisulfite Sequencing (RRBS) to establish differential DNA methylation targets in the path to colorectal cancer. RRBS has single-base resolution and detects CpG sites in gene bodies as well as intergenic regions, CpG islands and nonisland sequences, through highly sensitive sequencing of pooled sodium bisulfite modified CpG sequences [20]. The implementation of this technique within our study led to the identification of several potential markers that were selected based on the analysis of different combinations of samples (normal versus adenoma, adenoma versus cancer, and normal versus cancer). Here we identified specific CpG sites in* L3MBTL1, NKX6-2, PREX1, TRAF7, PRDM14*,and* NEFM* as primarily methylated in cancer specimens but not in other colonic lesions; these genes were also found to be implicated in many important pathways relevant to neoplastic transformation.

*L3MBTL1* gene located on chromosome 20q12 is a tumor suppressor gene. It is known to act as chromatin transcription repressor, which is activated mainly in germline stem cells [21]. This gene, which belongs to the polycomb group (PcG) proteins, binds to several methylated lysines in H1b, H3, and H4, blocking DNA sequences from access to transcription [21]. Zeng et al. found that high levels of* L3MBTL1* methylation were related to slightly elevated risk of breast cancer death, and hypermethylation of* L3MBTL1* in breast cancer patients can be reduced through physical exercise which positively associated with overall survival [21]. IPA analysis mapped this gene within a network of Wnt/*β*-catenin, P53, TGF-*β*, VEGF, P38 MAPK, and JAK/STAT3. In our study, this marker's methylation was noted in cancer but not adenoma samples which is in line with the type of pathways it is involved with through IPA analysis. To our knowledge, this is the first implication of this gene in colorectal cancer. If found in any preneoplastic lesions, this gene's methylation should point to highly carcinogenic lesions that need to be addressed and monitored aggressively due to its implication with major cancer pathways, including VEGF that points to metastasis implications as well as* NKX6-2* gene located on chromosome 10, known as a prognostic marker. Previous studies showed that* NKX6-2* is hypermethylated in bladder tumors and hypomethylated in normal leukocytes [22]. Another study demonstrated that DNA hypermethylation of* NKX6-2* is a hallmark of CIMP at very early stages of renal carcinogenesis [23].* NKX6-2* was also identified as a biomarker in stages II and III lung adenocarcinoma, where it was hypomethylated in stage II and hypermethylated in stage III [24]. The CpG sites from −538 to −575 upstream of* NKX6-2* are within the binding sites of SP1, SP3, and Egr-1 (Figure 1(b)) [25]. SP1 has been reported to serve as a transcriptional activator while SP3 acts as both an activator and a repressor, depending on cell and tissue type [25]. DNA methylation of promoter regions is associated with transcriptional silencing, either by preventing the binding of specific transcription factors or through the recruitment of methyl CpG binding proteins [25]. Egr-1 and SP1 are known to act as positive regulators of* NKX6-2*; this might elucidate the effect of these transcription factors on* NKX6-2 *gene [26]. IPA mapped this gene within the Wnt/*β*-catenin and P38 MAPK signaling pathways. We here report the first implication of this gene's methylation in colorectal carcinogenesis. The impact of the hypermethylated* NKX6-2* gene has been reported in bladder cancer, renal cancer, and lung adenocarcinoma, and this is consistent with our results in colorectal cancer specimens.

*TRAF7* is an E3 ubiquitin ligase for several proteins which is located in chromosome 16P. A study showed that downregulation of* TRAF7* is correlated with poor prognosis in breast cancer development [27]. IPA mapped this gene within a network of Wnt/*β*-catenin, P53, TGF-*β*, PI3K/AKT, VEGF, NF-k*β*, Hippo, P38 MAPK, and JAK/STAT3 signaling pathways. Here, we showed that hypermethylation of* TRAF7* is associated with cancer, but not preneoplastic specimens. To our knowledge this is the first time this gene is cited as a distinctive gene in CRC patients. The impact of the hypermethylated* TRAF7* gene has been reported in breast cancer and it is consistent with our results that this gene plays an important role in colorectal carcinogenesis.

*PRDM14* contains a PR domain that is likely a derivative of SET domain, which is involved in the methylation of lysine residues on the histone tail, and affects chromatin structure and gene expression [28]. A retrospective study showed that* PRDM14* is frequently overexpressed in breast cancers and that its overexpression is often associated with gene amplification [28]. We here report that this gene might also be a target of methylation in colorectal cancer. The present in silica study also suggests that DNA methylation of a CpG site located −620 to −627 bp upstream from the transcription start site in the promoter of PRDM14 may affect the expression of this gene [29]. CpG site −620 to −627 is located in the binding site to which NFY can bind (Figure 1(e)) [30]. Nuclear transcription factor Y (NFY) is a protein forming a highly conserved transcription factor in the promoter regions of a variety of genes. Previous studies suggested that NFY plays an important role in the regulation of genes that are expressed in various types of cancers and its involvement in tumor metastasis and breast cancer progression has been noted [30]. Moreover, NFY appears to regulate the expression of cell cycle and cell death related genes, suggesting its contribution to tumor cell proliferation [30]. IPA mapped this gene within a network of Hippo and P38 MAPK signaling pathways. The effect of the aberrant methylation of* PRDM14* gene has been reported in breast cancer and it is consistent with our results that hypermethylation of this gene plays an important role in many cancers.

*NEFM* is a gene located in chromosome 8p and is hypermethylated in renal cell carcinoma [31]. IPA mapped this gene within a network of Wnt/*β*-catenin, P53, TGF-*β*, PI3K/AKT, NF-k*β*, P38 MAPK, and JAK/STAT3 signaling pathways. To our knowledge this is the first time that* NEFM *is reported as a methylation target in colorectal cancer. The impact of the hypermethylated* NEFM* gene has been reported in renal cancer, and it is consistent with our results that this gene plays an important role in colorectal carcinogenesis. We are aware of the limitations of this study, such as the sample size, which is primarily due to the high costs associated with the RRBS methodology. As the technology becomes more accessible and cost effective, we plan larger size studies to validate the reported findings.

In conclusion, we here report six new distinct CpG methylation targets (*L3MBTL1, NKX6-2, PREX1, TRAF7, PRDM14*, and* NEFM*) in African Americans with CRC. These markers were found to be involved in major carcinogenic pathways and may be of a potential interest as CRC marker in African Americans.

## Figures and Tables

**Table 1:** Demographical characteristics of the analyzed samples.

Sample type	Gender	Age	Location
Normal blood	Male	60	NA
Normal colon tissue	Male	60	Right
Tubular adenoma	Male	59	Left
Tubulovillous adenoma	Female	76	Right
Tumor	Male	51	Left
Tumor	Female	69	Right
Tumor	Female	68	Right
Tumor	Male	55	Left
Tumor	Female	71	Left

NA: not applicable.

**Table 2:** Identified hypermethylated genes in colon neoplasia progression.

Genes	Spearman correlation	Adjusted *P* value (false detection rate)	Normal	Tubular adenoma	Tubulovillous adenoma	Tumor
*L3MBTL1*	0.22	2.93	0.0057	0.002353	0.00784	0.09
*NKX6-2*	0.08	0.000158	0.0144	0.00375	0.0000	0.12
*PREX1*	0.16	0.03	0.0057	0.0000	0.121	0.17
*TRAF7*	0.20	0.000244	0.0000	0.0000	0.0000	0.06
*PRDM14*	0.24	0.01	0.0000	0.0000	0.0000	0.27
*NEFM*	0.06	0.04	0.00	0.0000	0.0000	0.14

**Table 3:** Number of methylated CpG sites at promoter of identified genes.

Genes	Normal	Tubular adenoma	Tubulovillous adenoma	Tumor
*L3MBTL1*	0	0	0	22
*NKX6-2*	0	0	0	37
*PREX1*	0	0	6	27
*TRAF7*	0	0	0	17
*PRDM14*	0	0	0	28
*NEFM*	0	0	0	12

**(a):** 

Gene name	Crosstalk genes
*L3MBTL1*	*MMP3*
*NKX6-2*	*GCG*, *CREB1*
*PREX1*	*HDAC1*, *AKT1*
*TRAF7*	*IKBKB*
*PRDM14 *	*DLG4*
*NEFM*	*SMAD2*

**(b):** 

Pathways	Differentially methylated genes
Wnt/*β*-catenin	^*∗*^ *L3MBTL1, NKX6-2, * ^*∗*^ *PREX1,* ^*∗*^ *TRAF7, *and ^*∗*^ *NEFM*
P53	*L3MBTL1, PREX1, TRAF7, *and *NEFM*
TGF-*β*	*L3MBTL1, PREX1, TRAF7, *and *NEFM*
VEGF	*L3MBTL1, PREX1, TRAF7, *and *NEFM*
NF-k*β*	*PREX1, TRAF7, *and *NEFM*
Hippo	*PREX1, PRDM14, *and *NEFM*
JAK/STAT3	*L3MBTL1, PREX1, TRAF7, *and *NEFM*
P38 MAPK	*L3MBTL1, NKX6-2, PREX1, TRAF7, PRDM14, *and *NEFM*
PI3K/AKT	*L3MBTL1, PREX1, TRAF7, *and *NEFM*

^*∗*^Common pathways.
